# Effect of Plasticizers on Performance in Single-Ion Conducting Polymer Electrolytes: Implications for Lithium-Ion Batteries

**DOI:** 10.1021/acs.energyfuels.6c00725

**Published:** 2026-04-11

**Authors:** Linquan Gong, Adnan Al Najar, Anh Phan

**Affiliations:** School of Chemistry and Chemical Engineering, Faculty of Engineering and Physical Sciences, 3660 University of Surrey, Guilford, Surrey GU2 7XH, U.K.

## Abstract

Plasticizers play a crucial role in enhancing cation transport in single-ion conducting polymer electrolytes. However, a fundamental understanding of how different plasticizers facilitate ionic conductivity remains incomplete. To elucidate the molecular mechanisms by which plasticizers promote lithium-ion transport, equilibrium molecular dynamics simulations were performed to investigate lithium-ion transport behavior in modified polyethylene terephthalate (mPET) electrolytes plasticized with varying concentrations of fluoroethylene carbonate (FEC) and propylene carbonate (PC). Our simulation results show that both systems exhibit comparable lithium-ion diffusion coefficients and ionic conductivities at plasticizer concentrations below 40 wt %. In contrast, at concentrations above 40 wt %, the PC-plasticized system displays higher lithium-ion diffusion coefficients and ionic conductivities than the FEC-plasticized system. These observations can be attributed to the synergistic effects of plasticizer electrostatic properties and polymer chain flexibility. Specifically, quantitative comparisons of electrostatic surface potentials among FEC and PC indicate that the electron distribution of plasticizers governs their ability to compete with the polymer matrix for lithium-ion coordination, thereby determining the local coordination environment. In addition, radius of gyration analysis reveals that mPET chains in PC-plasticized systems exhibit greater flexibility, providing more continuous pathways for lithium-ion hopping between polymer chains. This enhanced flexibility is consistent with the reduced mean lifetimes of Li–O coordination pairs observed at plasticizer concentrations above 40 wt %. Overall, this work provides molecular-level insights that can guide the rational design of plasticizers to improve ionic conductivity in single-ion conducting polymer electrolytes.

## Graphical abstract

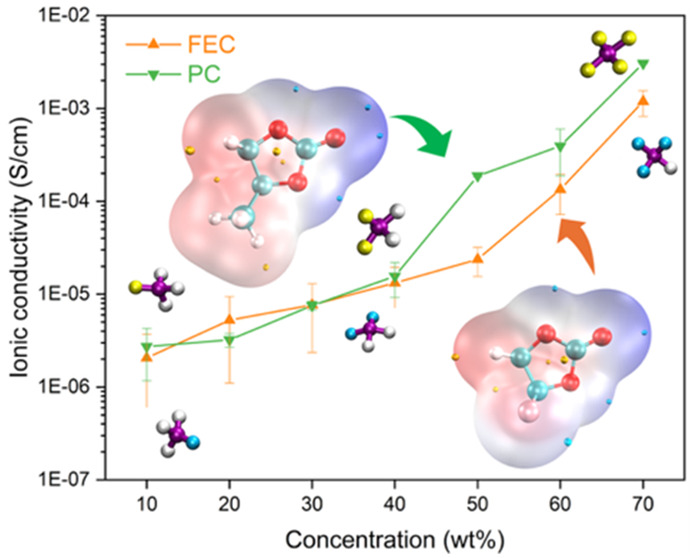

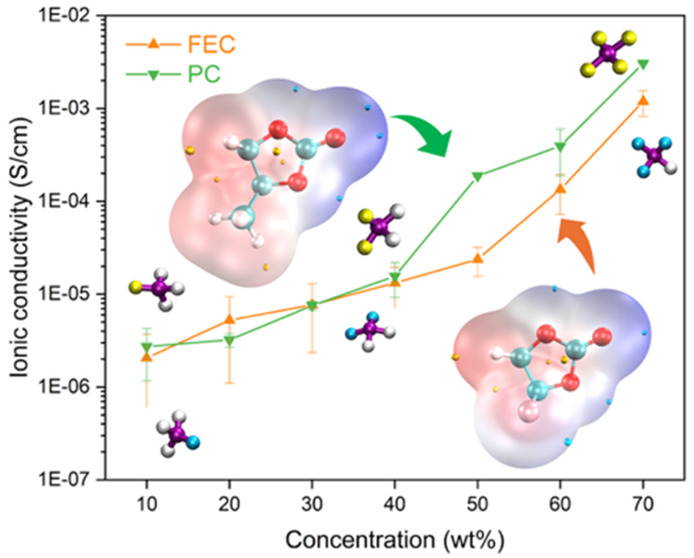

## 1 Introduction

Single-ion conducting polymer electrolytes (SICPEs) are widely recognized as one of the most promising electrolytes for the next-generation lithium batteries because of their lithium-ion transference number close to unity (*t*_Li+_ ≈ 1).^1–4^ In such systems, the anionic species are covalently tethered to the polymer backbone, such that only lithium ions act within charge transport. This molecular mechanism suppresses concentration polarization during electrochemical cycling, promotes uniform lithium deposition and mitigates the formation of electrochemically inactive ‘dead lithium’, therefore enhancing battery safety and cycle life.^5,6^ Despite these advantages, the practical industrial deployment of SICPEs has been hindered by their low ionic conductivity at ambient temperature, typically, several orders of magnitude lower than that of conventional liquid electrolytes.^7–10^ Nevertheless, this drawback can be alleviated through the addition of adequate amounts of small molecule plasticizers, frequently exhibit the ability to significantly diminish the crystallinity of polymer solid electrolytes and substantially enhance the ionic conductivity of SICPEs.^11–13^ The resulting synergy between improved electrochemical performance and enhanced safety makes SICPEs a highly attractive candidate for next-generation battery technologies. The plasticizers commonly employed in SICPEs are predominantly organic compounds, including ethylene carbonate (EC),^14^ propylene carbonate (PC),^14,15^ dimethyl carbonate (DMC),^16^ and diethyl carbonate (DEC).^17,18^

Chintapalli and Frech^19^ employed infrared spectroscopy in combination with conductivity measurements to investigate the influence of plasticizers—including EC, tetraethylene glycol, and tetraethylene glycol dimethyl ether—on the (PEO)­XLiCF_3_SO_3_ system. Their results demonstrated that plasticizers alter ionic association within polymer–salt complexes, leading to a pronounced enhancement in ionic conductivity. Buyting and Schönhoff^20,21^ used Raman spectroscopy, Nuclear Magnetic Resonance (NMR) titration, impedance spectroscopy, and pulsed-field-gradient NMR diffusion measurements to assess the effects of various cosolvents, such as sulfolane, dimethyl sulfoxide, and dimethylformamide, on the ion-transport properties of PEO/PEG-based electrolytes. They concluded that the observed conductivity enhancements primarily stem from modified Li–anion correlations in the presence of strongly Li-coordinating solvents. Fu et al.^22^ investigated the beneficial role of fluoroethylene carbonate (FEC) in an in situ polymerized composite electrolyte. They reported that increasing the FEC content raised the room-temperature ionic conductivity from 2.95 × 10–4 S cm^–1^ to 8.93 × 10–4 S cm^–1^, which was attributed to enhanced lithium–anion coordination in the solvent and a corresponding increase in lithium-ion mobility.

These experimental studies suggest that the primary effect of incorporating plasticizers is to alter lithium–anion interactions, thereby enabling the formation of ionically conductive pathways in SICPEs with dissolved ionic species. This, in turn, leads to enhanced lithium-ion diffusion coefficients and improved ionic conductivity. This interpretation is also supported by our previous simulation work^23,24^ on modified polyethylene terephthalate (mPET) with the addition of EC. However, current research has largely focused on screening different types of plasticizers, rather than elucidating the molecular mechanisms by which plasticizer molecular structure governs SICPE performance.^25–27^ A deeper mechanistic understanding is essential for developing a systematic, molecular-level design strategy for polymer electrolyte plasticizers in lithium-ion battery applications.

Like EC, both PC and FEC possess high relative permittivity and can therefore effectively promote the dissociation of ion pairs in polymer–salt complexes.^28^ Given their relatively minor structural differences compared to EC, an important question arises as to whether FEC and PC exhibit the same molecular mechanisms governing the performance of mPET–based SICPEs as previously reported for EC.^24^ To address this question, we employed equilibrium molecular dynamics simulations to elucidate the molecular-level mechanisms by which FEC and PC influence the performance of mPET-based SICPEs, with the aim of informing rational plasticizer design. By systematically comparing the effects of plasticizer type and concentration on lithium-ion mobility, local coordination environments, electrostatic potential distributions, and polymer flexibility, we seek to reveal how substituent chemistry modulates ionic conductivity in single-ion conductor systems and to establish a theoretical foundation for optimizing plasticizer structures from a molecular design perspective.

## 2 Simulation Methodology

### 2.1 Model Setup

Molecular models of the plasticizers (FEC and PC) and the mPET polymer were constructed using Moltemplate;^29^ their molecular structures are shown in Figure 1a and[Fig fig1]b, respectively. For the mPET model, the topology of the head, repeat unit, and tail segments must first be defined, along with the bonding atoms connecting these three fragments. Once these elements are specified, the complete model can be constructed. We investigated seven systems with plasticizer concentrations ranging from 10 to 70 wt % in 10 wt % increments. Detailed system compositions for each concentration are summarized in Table 1. We selected the number of mPET chains and corresponding PC/FEC molecules at each concentration to be consistent with the system sizes used in our previous study on EC-plasticized mPET-based systems. It is worth noting that the simulation results for the EC-plasticized systems are qualitatively consistent with the experimental data reported by Huu-Dat et al.^30^ and Dong et al.,^14^ which supports the reliability of the simulation protocol employed in the present study. Initial configurations were generated via PACKMOL,^31^ with representative snapshots of the 10 wt % FEC and PC systems illustrated in Figure 1c and 1e.

**Figure 1:**
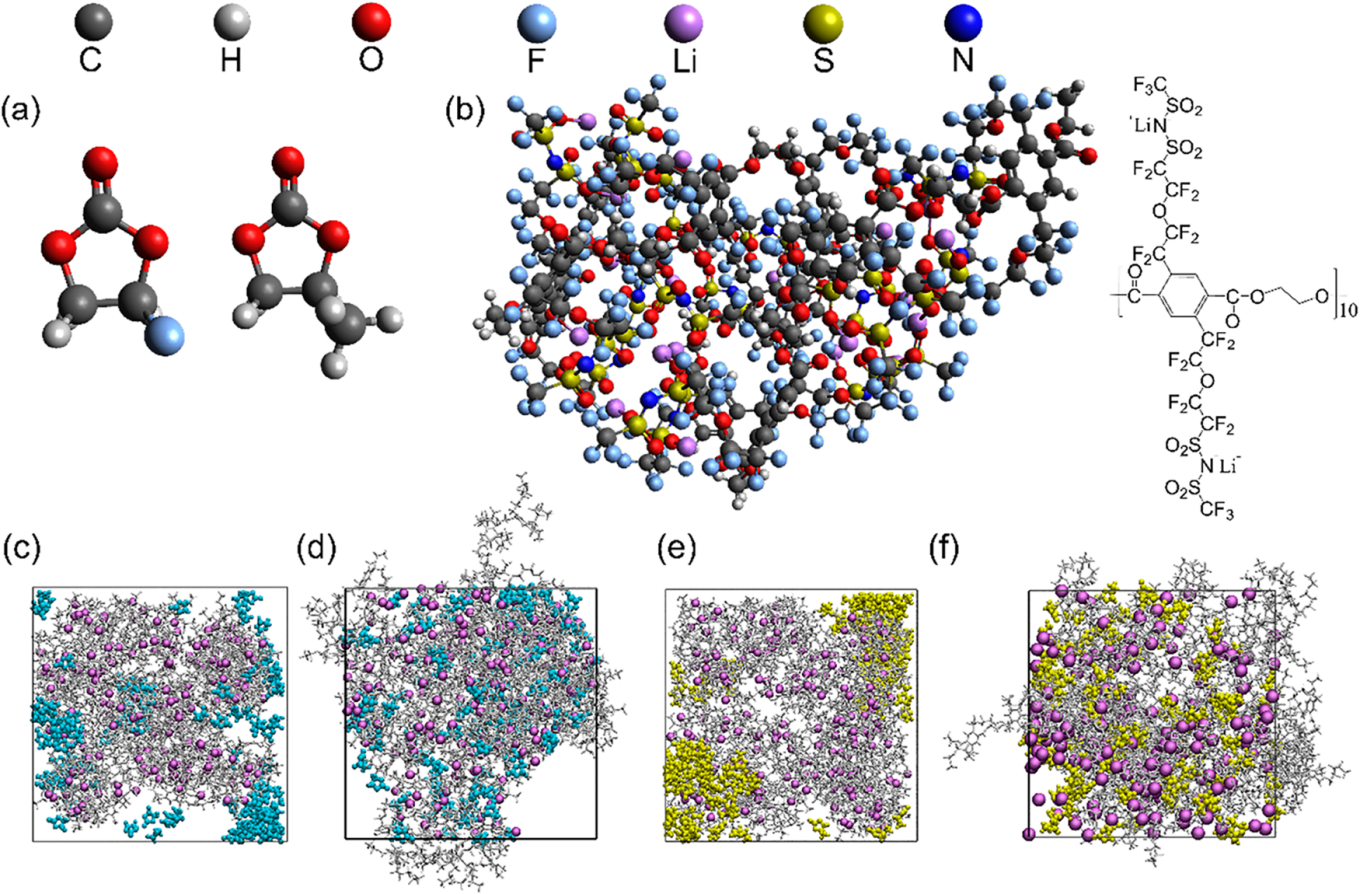
(a) Molecular structures of plasticizers FEC (left) and PC (right); (b) Optimized structure and molecular formula of a single mPET chain; (c, d) Initial and final equilibrated configurations for the 10 wt % FEC-plasticized system, where gray stick represents the mPET polymer, purple ball represents lithium ion, and cyan molecule represents FEC; (e, f) Initial and final equilibrated configurations for the 10 wt % PC-plasticized system, where gray stick represents the mPET polymer, purple ball represents lithium ion, and yellow molecule represents PC.

**Table 1:** Detailed System Compositions Considered in This Study

Concentration (wt %)	Number of mPET	Number of Li ions	Number of FEC	Number of PC
10	9	180	99	103
20	8	160	197	205
30	7	140	294	308
40	6	120	396	408
50	5	100	495	515
60	4	80	592	616
70	3	60	690	717

### 2.2 Force Fields

In our previous work,^24^ where the OPLS-AA force field was used to investigate the effect of EC concentration on the ionic and thermal conductivity of mPET-based SICPEs, the simulated ionic conductivities showed substantial deviations from experimental data at EC concentrations below 50 wt % (see Figure S1). This highlights the limitations of OPLS-AA in describing systems with pronounced polarization effects. In contrast, another study^25^ employing the generalized Amber force field (GAFF) combined with Restrained Electrostatic Potential (RESP) charges produced the results that were qualitatively consistent with experimental observations across all concentrations (see Figure S2). It is worth noting that ions exhibiting strong polarization behavior are present in the systems studied here, which can significantly influence local coordination structures. Therefore, to more accurately capture the relevant structure–property relationships, we have adopted the GAFF + RESP combination for the present simulations. Model parametrization was performed using Sobtop^32^ based on the GAFF,^33^ a framework widely validated for organic molecules and polymer systems.^34–37^ All molecular dynamics simulations were carried out using GROMACS (version 2021),^38^ applying the LINCS algorithm for hydrogen-bond constraints.^39^ Nonbonded interactions were described by 12–6 Lennard-Jones (LJ) potentials for dispersive forces and Coulombic potentials for electrostatic forces, with the latter utilizing Particle Mesh Ewald (PME) algorithm for long-range corrections.^40^ RESP charges^41^ were assigned to all species. Geometry optimizations for FEC and PC were performed at the B3LYP/6–311G­(d,p) level, while mPET was optimized at the B3LYP/6–311+G­(d,p) level. Subsequently, single-point energy calculations were executed at the B3LYP/def2tzvpp level to obtain high-precision electrostatic potentials. Further details on the RESP procedure are available in our previous work.^24^

### 2.3 Implementation

Each system followed a standardized equilibrium protocol. Initially, a single mPET chain with 20 lithium ions was heated to 1000 K and maintained for 3 ns under NVT ensemble, utilizing the velocity-rescale algorithm^42^ for temperature control. The system was subsequently cooled to 300 K under the NPT ensemble, with the Berendsen barostat^43^ maintaining the pressure at 1 bar. The resulting relaxed configuration (Figure 1b) and plasticizers were then packed into a periodic cubic box (*L* = 6 nm) according to the specified concentrations. To remove high-energy overlaps in the initial configurations, energy minimization was performed using conjugate gradient algorithm. The bulk systems then underwent the same heating and cooling cycle as the single mPET chain to ensure full relaxation. Following this, a 10 ns NPT equilibration was conducted using the same thermostats and barostats. Finally, the Nosé-Hoover thermostat^44^ and Parinello-Rahman barostat^45^ were employed to maintain the system at 300 K and 1 bar during a 100 ns production run, ensuring accurate volumes and densities, with the final 15 ns used for data sampling. Representative final configurations (10 wt % of FEC and PC) are shown in Figure 1d and 1f. Data analysis was performed using Multiwfn^46^ and custom Python scripts, while visualizations was generated by Visual Molecular Dynamics (VMD)^47^ and Avogadro.^48^

## 3 Results and Discussion

We first examined the dynamical properties of FEC- and PC-plasticized mPET-based SICPE systems as a function of plasticizer concentration. The results in Figure 2 show that the mean squared displacement (MSD) of lithium ions in both systems exhibits a positive correlation with increasing plasticizer concentration, following the similar trend as the EC-plasticized mPET systems reported in our previous study.^24^ Furthermore, we observed that the increase in MSD in the PC-plasticized system is more pronounced than that in the FEC-plasticized system, although both enhancements are less significant than those observed in the EC-plasticized system.

**Figure 2:**
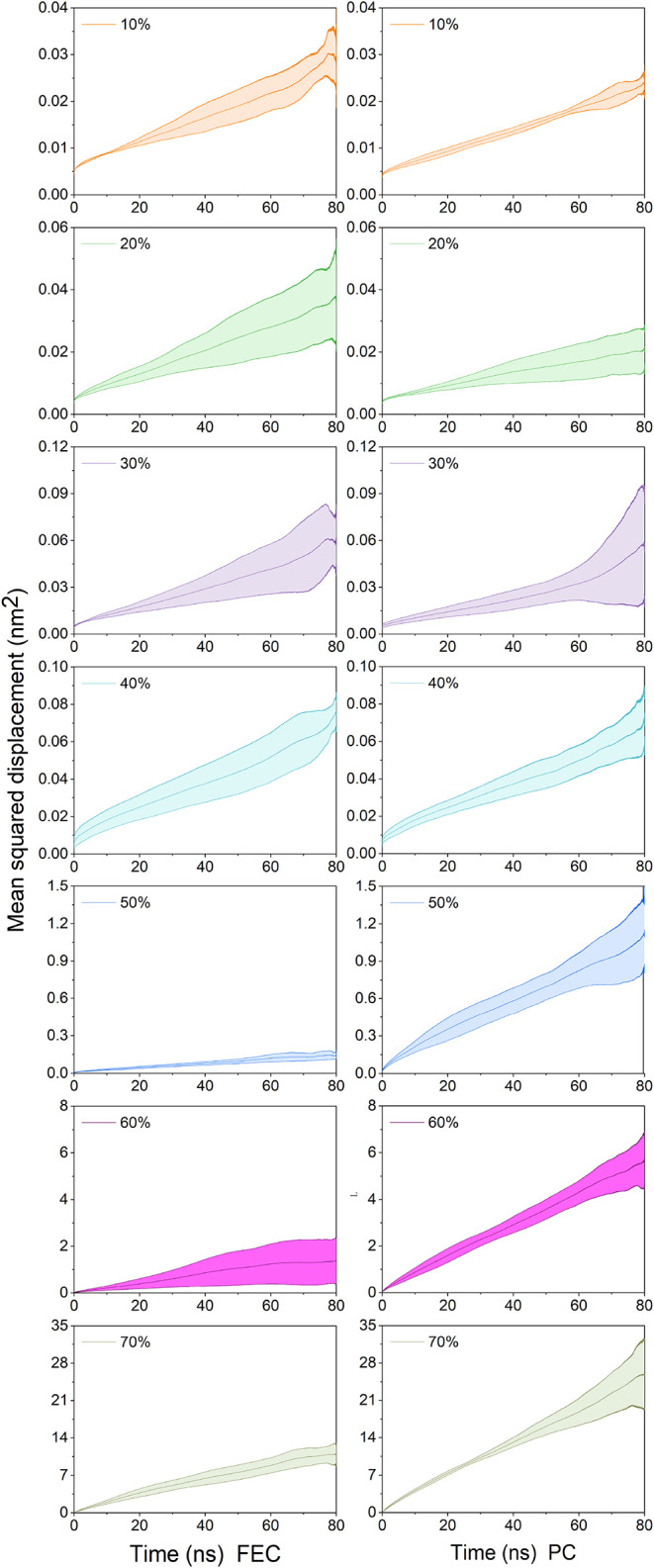
Mean squared displacements of lithium ions in FEC- (left panels) and PC-plasticized (right panels) systems across a concentration range of 10–70 wt %. Shaded areas denote the margin of error derived from three independent simulation runs for each system.

Lithium-ion diffusion coefficients and ionic conductivities were evaluated from the MSD data for both FEC- and PC-plasticized mPET-based SICPEs. The diffusion coefficients were obtained directly using the Einstein relation. To ensure reliable results, the nonlinear regions at the beginning and end of the MSD curves were excluded, and only the linear regime was used to determine the slope. As shown in Figure 3, both dynamical properties increase markedly with increasing plasticizer concentration. Specifically, as FEC concentration increases from 10 to 70 wt %, the lithium-ion diffusion coefficient rises from 3.13 × 10^–10^ to 3.57 × 10^–7^ cm^2^/s (Figure 3a) while the ionic conductivity increases from 2.06 × 10^–6^ S/cm to 1.19 × 10^–3^ S/cm (Figure 3b). For the PC-plasticized systems, the lithium-ion diffusion coefficient increases from 3.88 × 10^–10^ to 1.03 × 10^–6^ cm^2^/s (Figure 3a), with a corresponding rise in the ionic conductivity from 2.73 × 10^–6^ S/cm to 3.06 × 10^–3^ S/cm (Figure 3b). Notably, these properties increase only moderately and remain comparable between the two systems at concentrations below 40 wt %, where the choice of plasticizer exerts a negligible impact. However, increasing the EC content beyond 40 wt % leads to a markedly stronger enhancement in lithium-ion mobility. In this high-concentration regime, the PC-plasticized systems (green) exhibit higher diffusion coefficients and ionic conductivities than the FEC-plasticized ones (orange), demonstrating that PC is a more effective plasticizer for mPET-based SICPEs at elevated concentrations. We note that finite-size effects may influence the simulated properties. Nevertheless, we have employed the same simulation model setup for the PC- and FEC-plasticized mPET-based systems as in our previous work on EC-plasticized mPET-based systems, which showed good agreement with the experimental data reported by Huu-Dat^30^ and Dong et al.^14^ This consistency supports the reliability of the simulation protocol adopted in the present study.

**Figure 3:**
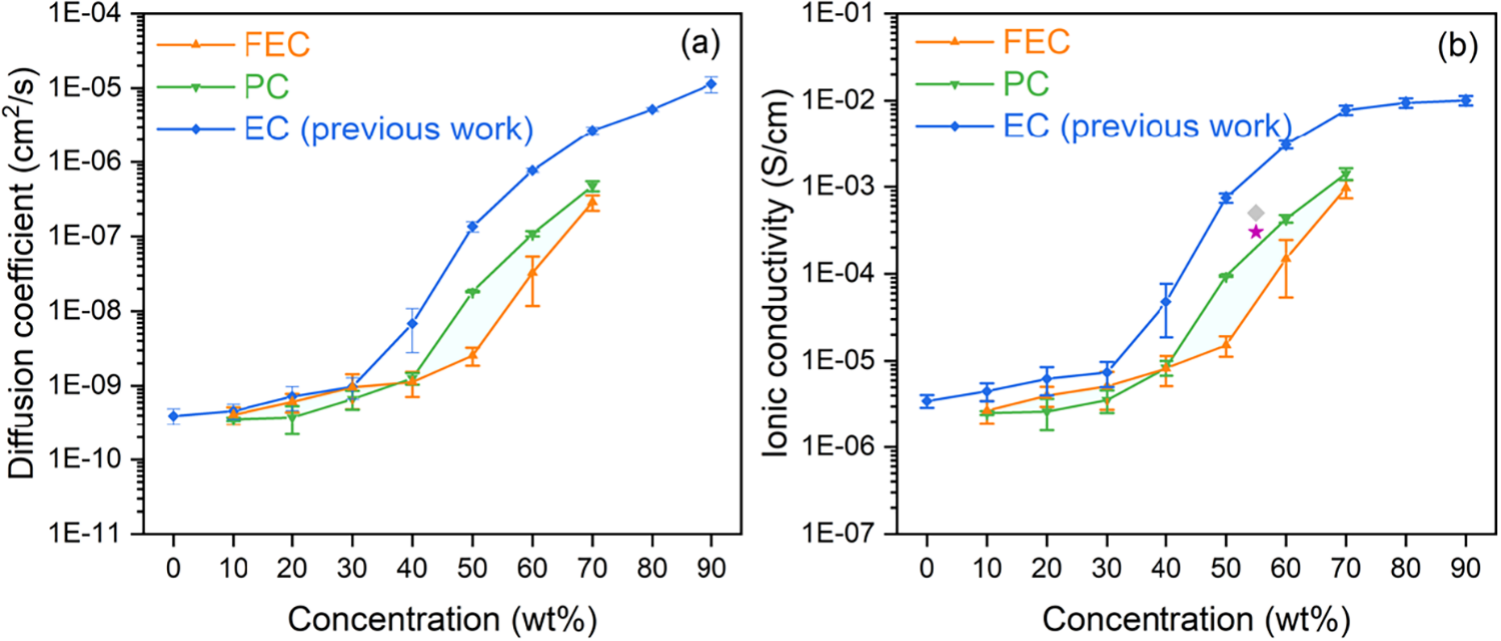
Concentration-dependent (a) diffusion coefficients of lithium ions and (b) ionic conductivities for mPET-based systems plasticized by FEC (orange) and PC (green) from 10 to 70 wt %. Results for systems plasticized by EC (blue), obtained in our previous work, are included for comparison (reproduced from Gong et al.^24^ Copyright 2025 by American Chemical Society). The gray diamond and purple star symbols represent the experimental ionic conductivity reported by Dong et al.^14^ at 55 wt % EC and EC-PC mixture for a structurally similar PBTFSI– system, respectively. Error bars represent the standard deviation derived from three independent simulations.

Dong et al.^14^ recently synthesized a novel poly­(arylene ether sulfone)-based SICPEs featuring two hydrogen-free side chains with a – SO_2_N–SO_2_CF_3_ structure (−(CF_2_)_2_O­(CF_2_)_2_SO_2_N^–^SO_2_CF_3_, known as PBTFSI^–^), which is structurally similar to our mPET-based SICPE model in this study. They experimentally examined the impact of plasticizers, such as EC and EC-PC mixtures, on the ionic conductivity across various temperatures. The lithium-ion conductivity reported by Dong et al.^14^ at 55 wt % EC (5 × 10^–4^ S/cm, gray diamond symbol in Figure 3b) is comparable to the simulated value extrapolated at a similar concentration (7.4 × 10^–4^ S/cm, blue one in Figure 3b) from our previous work.^24^ Furthermore, their study showed that the experimental ionic conductivity in the pure EC-plasticized system (gray diamond symbol) is higher than in the EC/PC mixture (purple star symbol) at the same temperature, indicating that the addition of PC lowers lithium-ion mobility. Notably, our simulation results demonstrate that the ionic conductivity of the pure PC-plasticized system (green) is lower than that of the pure EC-plasticized system (blue). This suggests that adding PC to an EC mixture would indeed reduce the overall ionic conductivity, which is qualitatively consistent with experimental observations. Moreover, the calculated ionic conductivities of EC-plasticized mPET-based systems at different concentrations in our previous work also show qualitative agreement with experiments (see Figure S2).^24^ These results further support the reliability of the force field parametrization used in this study.

It is well-established that the local coordination environment of lithium ions plays a critical role in determining the ionic conductivity of polymer electrolytes. Our previous study demonstrated that migration is strongly influenced by coordination structures within the mPET-based SICPEs containing EC as a plasticizer. However, a fundamental question remains regarding the quantitative analysis of SICPEs containing PC and FEC: Do PC and FEC plasticizers influence the mPET-based SICPEs in the same manner as EC?

To investigate this, we calculated the radial distribution functions (RDFs) and coordination numbers (CNs) between lithium ions and the oxygen atoms of both the plasticizers and mPET backbone across a range of concentrations (Figure 4). The Li–O pairs exhibit a primary peak at approximately 0.2 nm–regardless of whether the oxygen originates from the mPET backbone or the plasticizer–and a second peak near 0.4 nm. Additionally, a third smaller peak at 0.6 nm is observed specifically for Li–O_mPET_ interactions. As plasticizer concentration increases, the CN of Li–O_mPET_ decreases while the CN of Li–O_FEC_ or of Li–O_PC_ increases. This trend is consistent with our previous observations for EC-plasticized systems^24^ and suggests that higher concentrations of FEC or PC displace mPET oxygen atoms from the primary lithium-ion coordination shell (<0.3 nm). This shift toward plasticizer-dominated coordination enhances lithium-ion mobility and consequently, the overall ionic conductivities of mPET-based SICPEs, as illustrated in Figure 3. Furthermore, we observe that the CNs of Li–O_PC_ at concentrations above 40 wt % exceed those of Li–O_FEC_. For instance, the CN of Li–O_PC_ at 50 wt % PC within the first coordination shell (<0.3 nm) is 2.35, which is greater than that of Li–O_FEC_ at same concentration (1.89). This observation aligns with the higher lithium-ion diffusion coefficients and ionic conductivities observed at plasticizer concentration exceeding 40 wt %.

**Figure 4:**
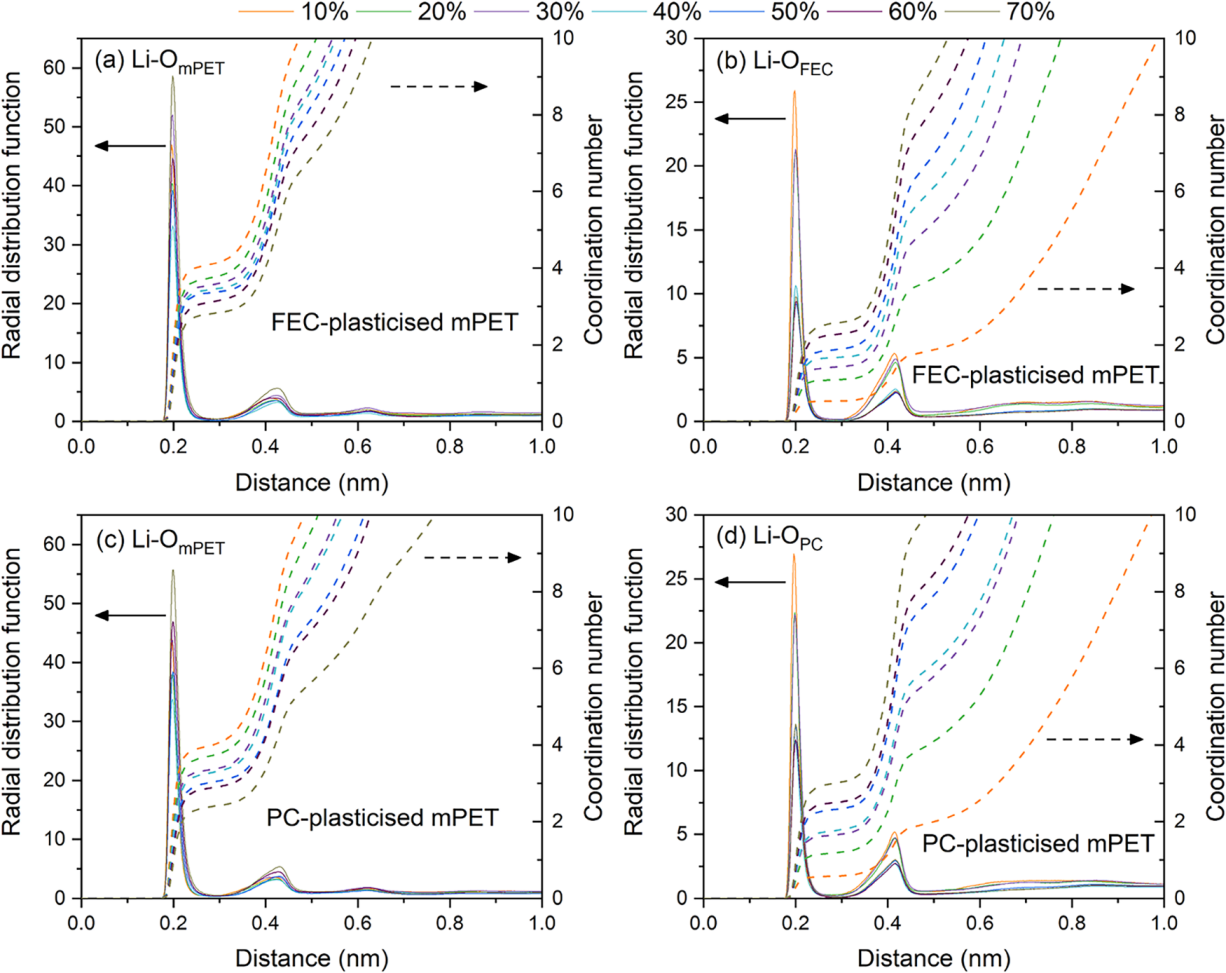
Radial distribution functions (RDFs) and corresponding coordination numbers (CNs) for Li–O pairs involving the mPET backbone (left panels) and plasticizers (right panels) at concentrations from 10 to 70 wt %. Data are shown for FEC-plasticized (a, b) and PC-plasticized mPET-based SICPEs (c, d).

To gain further insight into the local coordination environment of lithium ions, we analyzed the configurations of lithium ions coordinated with oxygen atoms from mPET and the plasticizers (FEC and PC) within the first coordination shell (<0.3 nm) at various concentrations (see Figure 5a). At plasticizer concentrations below 40 wt %, the number of coordinating mPET oxygen atoms is higher than that of either FEC or PC. However, with further increases in plasticizer concentration, the number of coordinating mPET oxygen atoms decreases. To quantify the effects of plasticizer concentration and type on lithium-ion transport, we calculated the ratio of oxygen atoms from the plasticizer to the total number of coordinating oxygen atoms from both mPET and the plasticizer within the first lithium-ion coordination shell (Figure 5b). In the PC-plasticized system, this ratio increases from 0.13 to 0.50 as the plasticizer concentration rises from 10 to 70 wt % (yellow), whereas in the FEC-plasticized system it increases from 0.11 to 0.39 (blue) over the same concentration range. Notably, the ratio is consistently higher in the PC-plasticized system than in the FEC-plasticized system at all concentrations, with the difference becoming more pronounced above 40 wt %, in agreement with the trends observed in the lithium-ion diffusion coefficients and ionic conductivities shown in Figure 3. Importantly, in both FEC- and PC-plasticized systems, all ratios remain ≤ 0.5 across the entire concentration range, indicating that the first coordination shell is still predominantly governed by mPET oxygen atoms. This implies that the differences in lithium-ion diffusion coefficients and ionic conductivities between FEC- and PC-plasticized systems arise from the combined effects of Li–O_mPET_ and Li–O_FEC/PC_ interactions rather than from plasticizer coordination alone.

**Figure 5:**
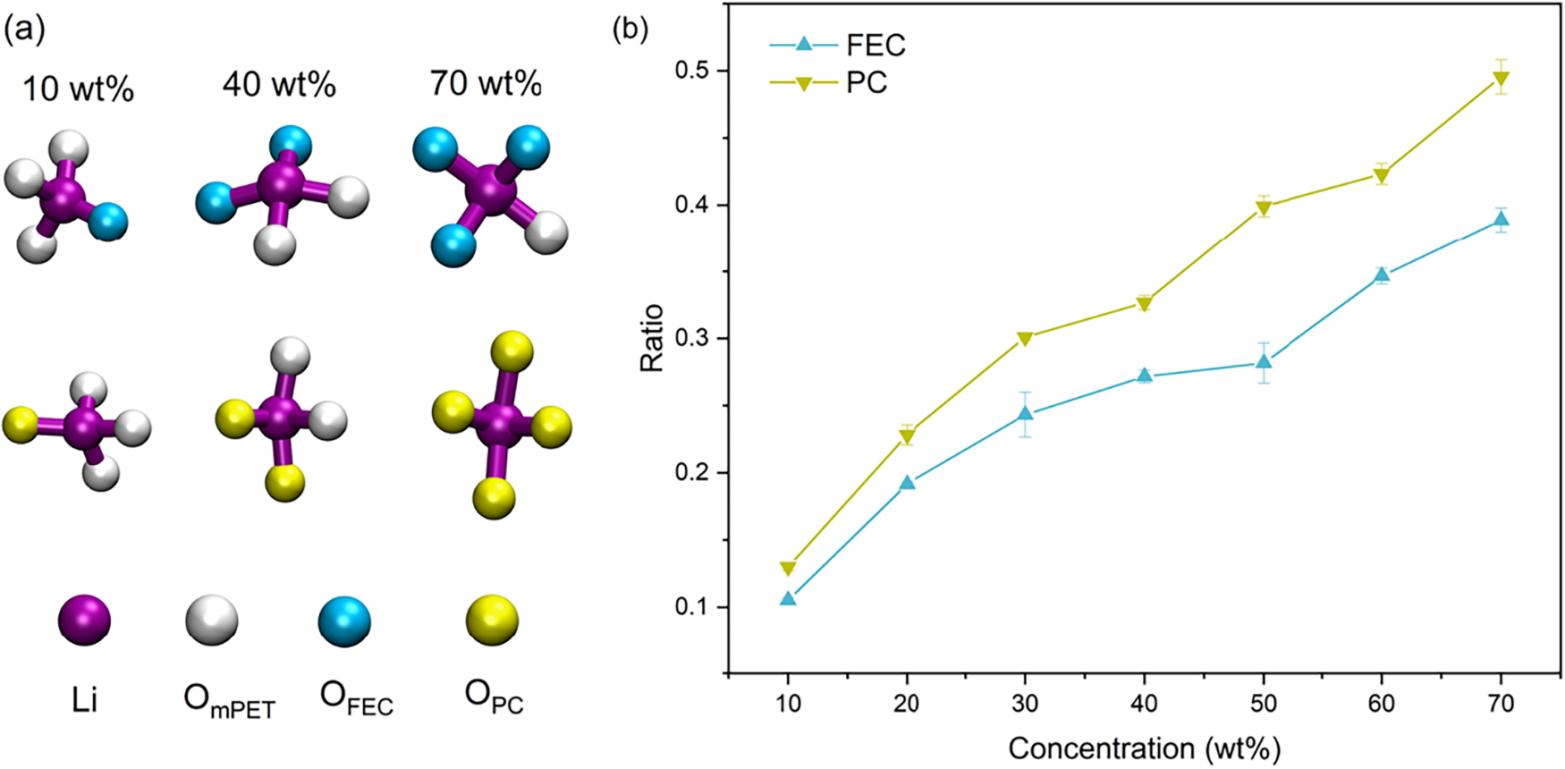
(a) Representative coordination structures illustrating the lithium ions (purple) coordinated with oxygen atoms from mPET (white) and the plasticizers—FEC (cyan) and PC (yellow)—within the first coordination shell (<0.3 nm) at 10, 40, and 70 wt % concentrations. (b) Ratio of oxygen atoms from FEC (blue) or PC (yellow) to the total number of coordinating oxygen atoms from both mPET and the plasticizer within the first lithium-ion coordination shell for all systems.

To better elucidate the synergistic effects of mPET and plasticizers on the correlation between the dynamical properties and the local coordination environment of lithium ions, we evaluated the dissociation behavior of lithium ions from mPET and the plasticizers by computing the mean lifetimes of Li–O_mPET_ and Li–O_FEC/PC_ (FEC or PC) pairs (Figure 6). The results show that the mean lifetimes of both Li–O_mPET_ and Li–O_FEC/PC_ pairs generally decrease with increasing plasticizer concentration in both FEC- and PC-plasticized systems, exhibiting a rapid decline at low concentrations followed by a more gradual decrease at higher concentrations. Notably, the mean lifetime of Li–O_mPET_ pairs is approximately 2 orders of magnitude longer than that of Li–O_FEC/PC_ pairs, indicating that Li–O_mPET_ interactions predominantly govern the hopping of lithium ions out of the first coordination shell. As the ratio of oxygen atoms from FEC or PC to the total number of coordinating oxygen atoms from both mPET and the plasticizer increases with plasticizer concentration, lithium ions can more readily escape the first coordination shell at higher plasticizer contents, thereby enhancing lithium-ion mobility.

**Figure 6:**
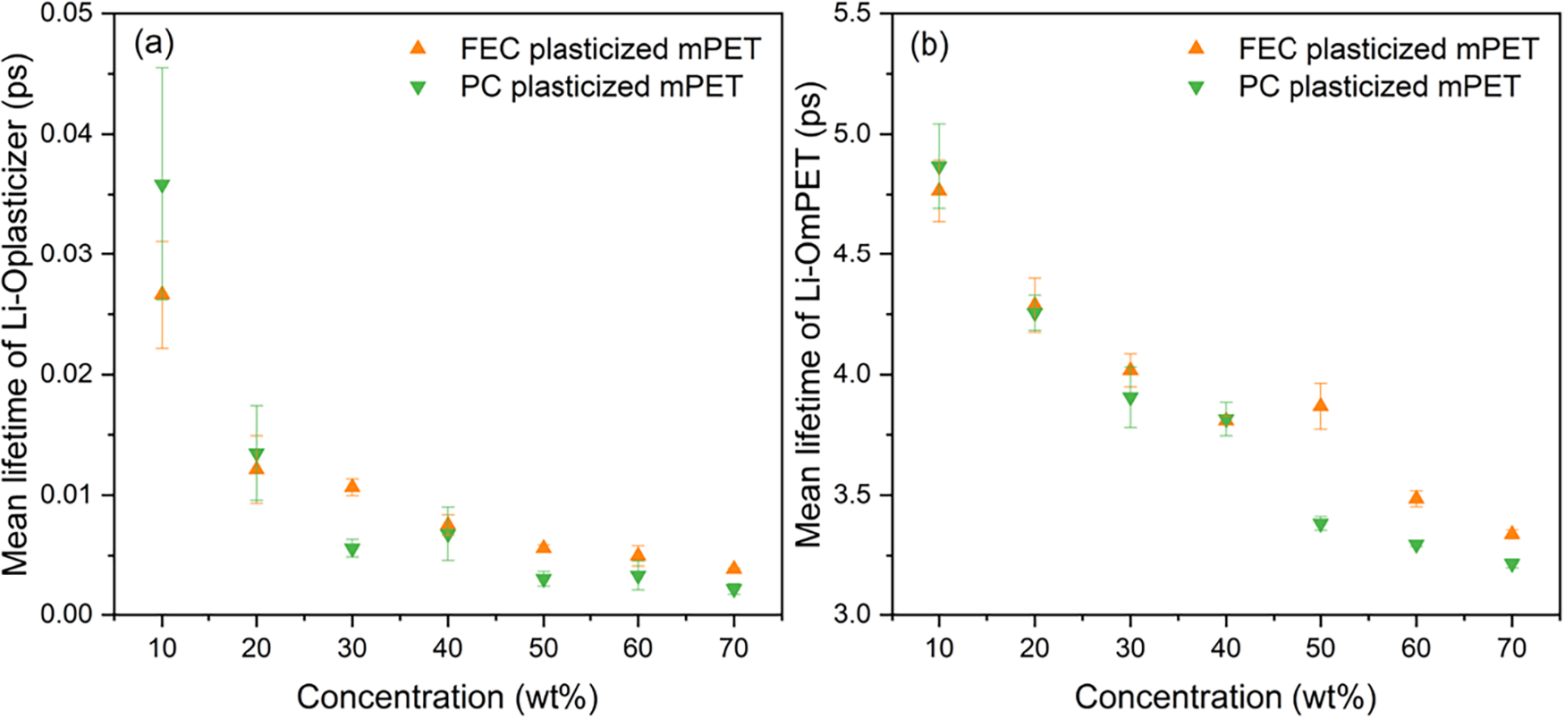
(a) Mean lifetimes of Li–O_FEC/PC_ and (b) Li–O_mPET_ pairs as a function of concentration. Results are shown for FEC-plasticized (orange) and PC-plasticized (green) mPET-based SICPEs.

Furthermore, at plasticizer concentrations above 40 wt %, the mean lifetimes of Li–O_PC_ pairs (green, Figure 6a) are slightly shorter than those of Li–O_FEC_ pairs (orange, Figure 6a), while the mean lifetimes of Li–O_mPET_ pairs in the PC-plasticized systems (green, Figure 6b) are markedly shorter than those in the corresponding FEC-plasticized systems (orange, Figure 6b). This suggests that PC is more effective than FEC at disrupting lithium–mPET interactions at high concentrations, which may contribute to the observed differences in lithium-ion diffusion coefficients and ionic conductivities above 40 wt %. In contrast, at concentrations below 40 wt %, the mean lifetimes of Li–O_mPET_ pairs show negligible differences between the FEC- and PC-plasticized systems. These observations at low concentrations are likely because neither FEC nor PC effectively plasticizes mPET below 40 wt %. As a result, the plasticized mPET-based SICPEs do not yet exhibit sufficiently liquid-like behavior to produce pronounced differences in lithium-ion mobility.

To gain a deeper understanding of how the molecular structures of different plasticizers influence lithium-ion mobility, we compared the electrostatic potential (ESP) distributions of FEC and PC, with EC included for reference. The molecular ESP describes the electrostatic interaction energy between a unit positive test charge and the charge distribution of a molecule, accounting for contributions from both nuclei and electrons. Regions with negative ESP correspond to electron-rich areas that are favorable for interactions with cationic species, whereas regions with positive ESP indicate electron-deficient areas that tend to repel cations. Consequently, negative ESP regions are expected to preferentially interact with lithium ions via electrostatic attraction and thus represent potential coordination sites.^49^

Quantitative analysis reveals that the EC molecule exhibits two equivalent ESP minima (blue points, −38.72 kcal/mol; Figure 7, left panel) located near the carbonyl oxygen atom. In contrast, fluorine in FEC significantly alters the electron distribution relative to EC, resulting in four ESP minima at distinct sites (−19.45, −34.60, −20.75, and −14.74 kcal/mol; Figure 7, middle panel), while reducing the magnitude of the most negative minimum. This redistribution of electron density suggests that FEC is less effective at competing with mPET for lithium-ion coordination than EC. In comparison, the ESP distribution of PC does not exhibit a substantial rearrangement of electron density. Instead, two additional ESP minima appear near either side of the single-bond oxygen atom (−26.65 and −25.91 kcal/mol; Figure 7, right panel). Moreover, the minimum ESP value of PC (−40.34 kcal/mol) is more negative than that of FEC (−34.6 kcal/mol) indicating that PC has a stronger ability to coordinate with lithium ions than FEC. This comparative ESP analysis provides a molecular-level explanation for the greater number of oxygen atoms from PC coordinating with lithium ions within the first coordination shell than that from FEC, resulting in the larger ratio reported in Figure 5. It is worth noting that the electrostatic potential distributions of FEC, PC and EC obtained in this study are consistent with those reported by Wu et al.^50^ and Ying et al.,^51^ supporting the reliability of the ESP analysis presented here.

**Figure 7:**
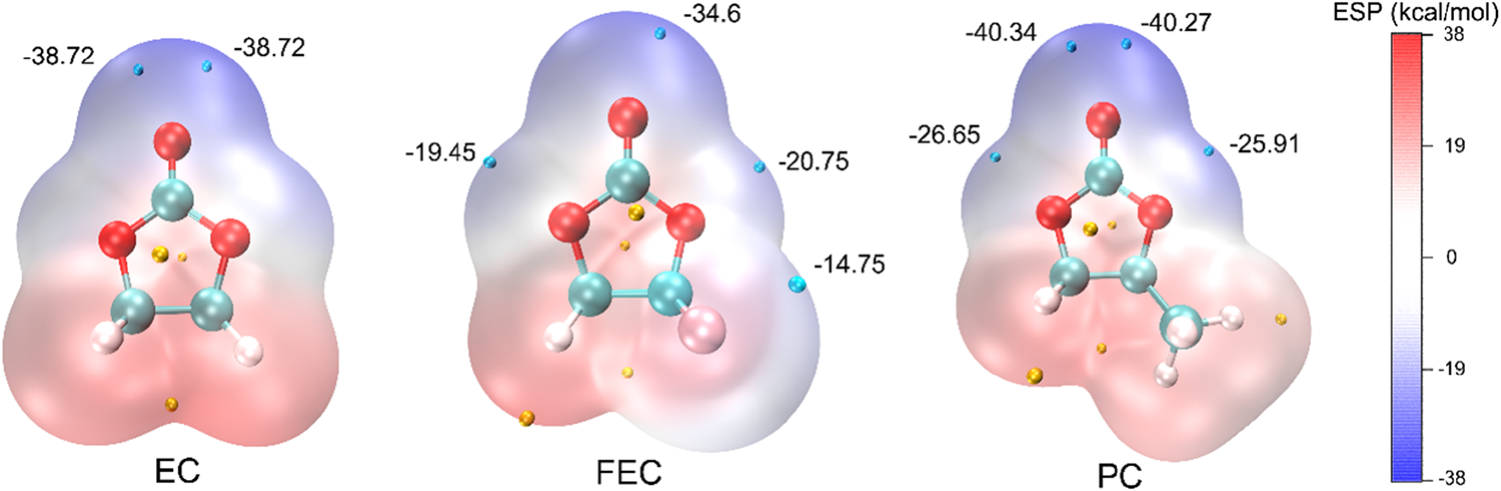
Electrostatic potential (ESP) distributions of FEC and PC, with EC included for reference, where blue points correspond to minimums and yellow point correspond to maximums.

Although the ESP analysis explains why PC exhibits a stronger ability to coordinate with lithium ions than FEC, variations in plasticizer type alone cannot fully account for the differences in lithium-ion mobility observed between FEC- and PC-plasticized systems. We therefore examine the radius of gyration of mPET chains in both systems, which reflects polymer chain compactness—a key factor governing lithium-ion transport through mPET matrices.

A larger radius of gyration indicates enhanced polymer chain flexibility and the presence of more continuous transport pathways, which are generally favorable for lithium-ion migration, as ions can hop more readily between polymer chains. In Figure 8a, we present representative equilibrium configurations of mPET chains in FEC- and PC-plasticized systems at 10, 40, and 70 wt %. In both systems, mPET chains are highly compact at 10 wt % plasticizer and become progressively less compact with increasing plasticizer concentration at 40 and 70 wt %. These visual observations suggest that the radius of gyration of mPET increases with increasing plasticizer concentration.

**Figure 8:**
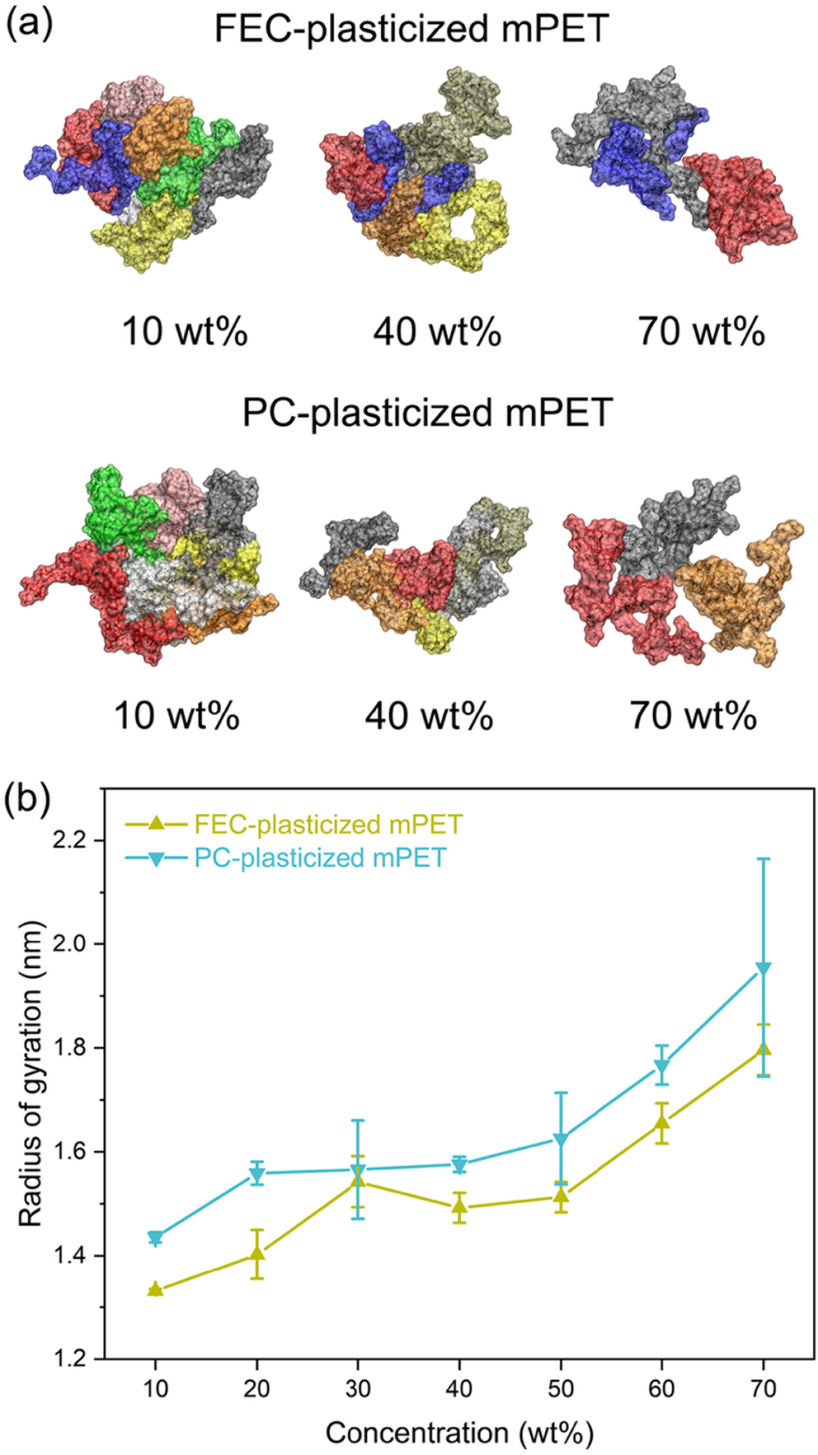
(a) Representative equilibrium configurations of mPET chains in the FEC- and PC- plasticized systems at 10, 40, 70 wt %. (b) Average radius of gyration of mPET chains as a function of plasticizer concentration.

To quantitatively confirm this trend, we computed the radius of gyration at plasticizer concentrations ranging from 10 to 70 wt % for both systems (Figure 8b). The results show that the radius of gyration of mPET increases with plasticizer concentration in both cases. Specifically, the radius of gyration of mPET increases from 1.33 to 1.80 nm as the FEC content increases from 10 to 70 wt %, while it increases from 1.44 to 1.95 nm as the PC content increases over the same concentration range. At concentrations above 40 wt %, the radius of gyration of mPET in the PC-plasticized system is consistently higher than that in the FEC-plasticized system, in line with the trends in lithium-ion diffusion coefficients and ionic conductivities (see Figure 3). At lower concentrations (10–20 wt %), the radius of gyration is also slightly higher for PC-plasticized mPET, while similar values are observed at 30 wt %. It is worth noting that neither FEC nor PC sufficiently plasticizes mPET below 40 wt %, and the resulting mPET-based SICPEs do not yet display adequately liquid-like behavior to yield significant differences in lithium-ion mobility.

Overall, lithium-ion transport is governed by the synergistic effects of plasticizer electrostatic interactions and polymer flexibility. The ESP of the plasticizers determines their ability to compete with mPET for lithium-ion coordination, increasing the probability of lithium ions coordinating with plasticizer oxygen atoms. This shift in coordination reduces the mean lifetime of Li–O_mPET_ pairs as plasticizer concentration increases, thereby facilitating lithium-ion transport. At the same time, a larger radius of gyration of mPET creates more continuous pathways for lithium-ion hopping between polymer chains. Notably, at plasticizer concentrations above 40 wt %, the enhanced flexibility of mPET—due to its greater sensitivity to plasticizer content—leads to a pronounced improvement in lithium-ion mobility.

## 4 Conclusion

We employed equilibrium molecular dynamics simulations to investigate the effects of FEC and PC concentrations on lithium-ion mobility. The results show similar lithium-ion diffusion coefficients and ionic conductivities at plasticizer concentrations below 40 wt % for both systems. At concentrations above 40 wt %, however, PC-plasticized systems exhibit significantly higher lithium-ion diffusion coefficients and ionic conductivities than FEC-plasticized systems. Analysis of the ratio of Li–O_FEC/PC_ coordination pairs to the total number of Li–O_FEC/PC_ and Li–O_mPET_ pairs, together with the mean lifetimes of these coordination pairs within the first solvation shell of lithium ion, indicates that lithium-ion transport is governed by local coordination structures and the ability of lithium ions to escape their coordination shells. Quantitative electrostatic potential (ESP) analysis further reveals that the local coordination structures depend on the electron distribution of the plasticizers: plasticizers with more negative ESP minima exhibit stronger coordination with lithium ions. In addition, analysis of the radius of gyration of mPET chains in both plasticized systems confirms that polymer flexibility plays a critical role in lithium-ion mobility. Specifically, at plasticizer concentrations above 40 wt %, mPET chains in the PC-plasticized system become more flexible, facilitating faster interchain lithium-ion transport and resulting in higher ionic conductivity. Collectively, these results demonstrate that lithium-ion transport is jointly determined by the electronic characteristics of the plasticizers and the flexibility of the polymer matrix. This work provides guidance for optimizing plasticizer selection to enhance ionic conductivity in single-ion conducting polymer electrolytes by simultaneously considering plasticizer electron distribution and polymer flexibility.

## Supplementary Material





## Notes

Details on the comparison of lithium-ion conductivity between simulated and experimental results, as well as the increase in the mean squared displacement of lithium ions across the plasticizer concentration range (PDF)
Input files and final equilibrated trajectory files generated in this study (ZIP)
