# Supplementary material for: Effect of Plasticizers on Performance in Single-Ion Conducting Polymer Electrolytes: Implications for Lithium-Ion Batteries
Source: Energy Fuels. 2026 Apr 11;40(16):9105–14. doi: 10.1021/acs.energyfuels.6c00725 (PMC13112336; doi:10.1021/acs.energyfuels.6c00725)
Supplement: Supplementary file 1 [file ef6c00725_si_001.pdf]

Supporting Information

Effect of Plasticizers on Performance in Single-Ion Conducting Polymer Electrolytes: Implications for Lithium-Ion Batteries

Linquan Gong*, Adnan Al Najar, Anh Phan*

School of Chemistry and Chemical Engineering, Faculty of Engineering and Physical Sciences, University of Surrey, Guilford, Surrey GU2 7XH, UK

*Corresponding Authors: l.gong@surrey.ac.uk, a.phan@surrey.ac.uk

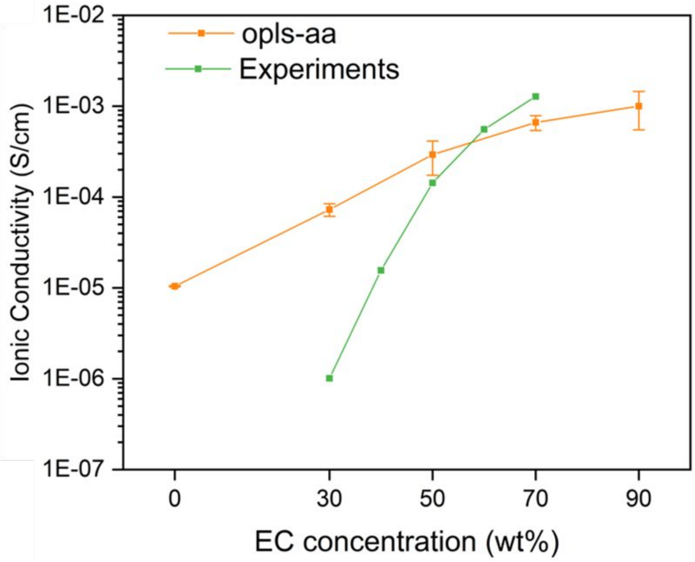

Figure S1. Comparison of lithium ionic conductivity between simulated results[1] and experimental data reported by Nguyen et al[2]. Reproduced from Gong et al.[1] Copyright 2025 by Elsevier.

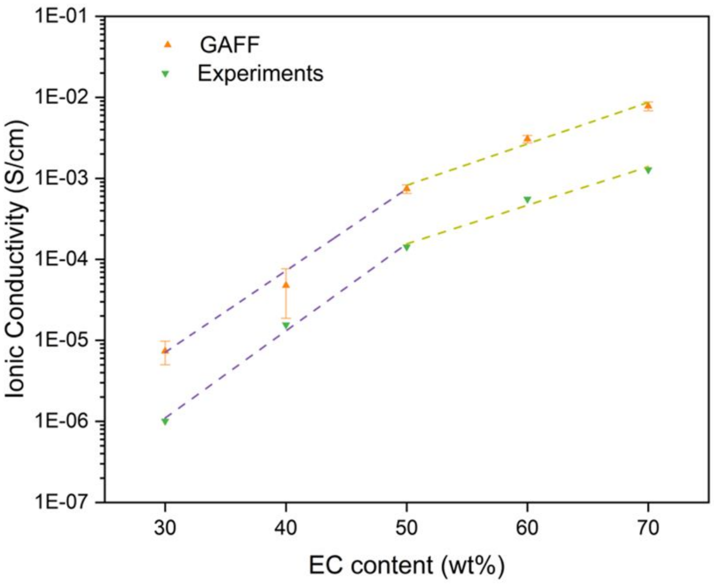

Figure S2.Comparison of lithium ionic conductivity between simulated results[3] and experimental data reported by Nguyen et al[2]. Reproduced from Gong et al.[3] Copyright 2025 by American Chemical Society

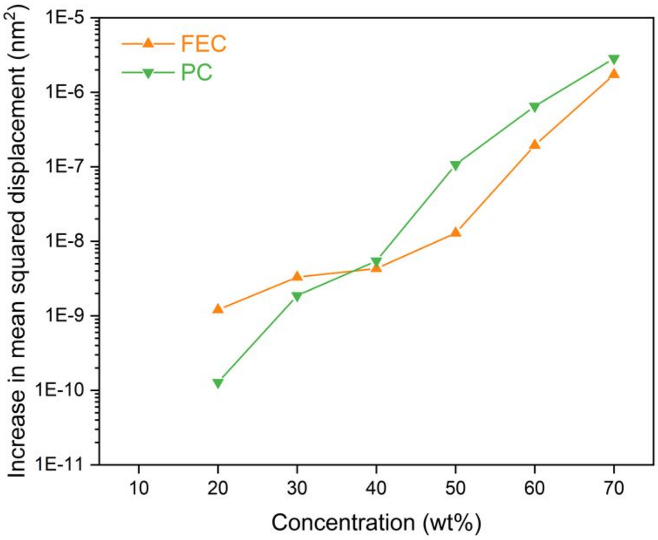

Figure S3. Increase in the mean squared displacement of lithium ions in FEC- (orange) and PC-plasticised (green) systems over a concentration range of 10–70 wt%.

References

(1) Gong, L.; Gadkari, S.; Pan, Y.; Phan, A. Factors governing ionic and thermal conductivity of plasticized single-ion conducting polymer electrolytes: A molecular perspective. *Chemical Engineering Journal* **2025**, *525*. DOI: 10.1016/j.cej.2025.170124.

(2) Nguyen, H.-D.; Kim, G.-T.; Shi, J.; Paillard, E.; Judeinstein, P.; Lyonnard, S.; Bresser, D.; Iojoiu, C. Nanostructured multi-block copolymer single-ion conductors for safer high-performance lithium batteries. *Energy & Environmental Science* **2018**, *11* (11), 3298-3309. DOI: 10.1039/c8ee02093k.

(3) Gong, L.; Gadkari, S.; Pan, Y.; Phan, A. Molecular Mechanisms Driving the Performance of Single-Ion Conducting Polymer Electrolytes in Lithium-Based Batteries. *Langmuir* **2025**. DOI: 10.1021/acs.langmuir.5c00735.
